# Macrophage phagocytosis of *Coccidioides* promotes its differentiation into the parasitic form

**DOI:** 10.1128/mbio.00492-26

**Published:** 2026-05-18

**Authors:** Jane Symington, Mark Voorhies, Bevin C. English, Apoorva Dabholkar, Anita Sil

**Affiliations:** 1 Department of Microbiology and Immunology, University of California San Francisco 8785, San Francisco, California, USA; 2 Department of Pediatrics, University of California San Francisco 8785, San Francisco, California, USA; 3 CoLabs, University of California San Francisco 8785, San Francisco, California, USA; 4 Biohub 578083 , San Francisco, California, USA; Instituto Carlos Chagas, Curitiba, Brazil

**Keywords:** fungal pathogenesis, macrophages, fungal development, Valley Fever

## Abstract

*Coccidioides* is an endemic fungus that is increasing in prevalence and causes life-threatening diseases in immunocompetent people. In the environment, the spores (arthroconidia) develop into hyphae; however, when they are inhaled by a mammalian host, they develop into a unique form called the spherule. The transition to spherule can be triggered *in vitro* with elevated temperatures and high CO_2_ levels, but how the host triggers *Coccidioides* spherulation is not known. We used live imaging to investigate how macrophages affect the fate of *Coccidioides* arthroconidia. Under tissue culture conditions, arthroconidia quickly developed into hyphae. Remarkably, the addition of macrophages promoted spherule development and delayed hyphal formation. Macrophage supernatants were not sufficient to promote spherule development, and chemical blockade of phagocytosis inhibited spherule formation. Transcriptomics analysis of *Coccidioides* co-cultured with macrophages revealed a signature concordant with spherules grown *in vitro* and allowed the identification of a core set of 143 spherule-specific transcripts. Additionally, we identified 229 *Coccidioides* transcripts with significantly higher abundance in the presence of macrophages compared to *in vitro* generated spherules, suggesting that these factors may be needed for growth in the presence of innate immune cells; 48 induced transcripts were predicted to encode secreted proteins, suggesting a function at the host-pathogen interface. Taken together, this work highlights the capacity of macrophages to promote development of the parasitic form of *Coccidioides* and lays a foundation for uncovering host-pathogen signaling as well as *Coccidioides* factors that are critical for pathogenesis.

**IMPORTANCE:**

Valley Fever is a disease caused by inhalation of the spores of the fungus, *Coccidioides* spp. It can present like the flu, pneumonia, bone infections, or meningitis. Once inhaled, the spores change into a pathogenic form that allows the fungus to spread throughout the body and cause disease. How the spores make this transition in the body is not well understood. We investigated how immune cells affected this transition. We found that engulfment of spores by innate immune cells stimulated the transition to the pathogenic form of the fungus. We determined which fungal genes are induced during interactions with innate immune cells, potentially identifying genes that may be critical for the development of the pathogenic form. This work helps us understand how this pathogen is taking advantage of our immune system to survive and cause disease.

## INTRODUCTION

*Coccidioides* spp. are fungal pathogens that cause Coccidioidomycosis or Valley Fever in both immunocompetent and immunocompromised individuals. Although many experience a self-limiting disease, others develop severe disseminated forms, including meningitis, which requires lifelong therapy (1). Coccidioidomycosis cases are rising nationally, and the endemic area may be spreading, making better diagnostics and therapeutics essential. Our understanding of this pathogen is limited in part because *Coccidioides* has a unique parasitic morphological form called the spherule. In the environment, *Coccidioides* grows as hyphae, but when the spores (arthroconidia) are inhaled, they undergo a morphologic transition to the spherule, which can grow as large as 40–100 μm in diameter. The spherule is filled with hundreds of internal cells called endospores (2), which are released upon spherule rupture. Each endospore can develop into a spherule and further spread the disease.

Our understanding of spherule development is largely based on *in vitro* experiments with specialized media, elevated temperatures (39°C), and elevated CO_2_ (~10%–20%) (3, 4). These studies have produced key understandings of the timing of events in the development of spherules from arthroconidia (5, 6); however, how well these *in vitro* studies mimic events within a mammalian host remains unknown. Many open questions about *Coccidioides*-host interactions remain, including how the transition to spherules is triggered in the host and how arthroconidia avoid clearance by innate immune cells.

The initial infection site with *Coccidioides* is the lung. Respiratory pathogens like *Coccidioides* must overcome the host defenses of the lung, including macrophages, other immune cells, and epithelial cells, to cause disease. Many successful respiratory pathogens, including *Histoplasma capsulatum* and *Mycobacterium tuberculosis,* subvert anti-microbial processes in macrophages to survive in the host (7, 8). Early papers suggested that a variety of immune cells could kill *Coccidioides* arthroconidia, delay germination of hyphae, or possibly promote spherulation (9–11). Here, we take advantage of live-cell imaging in a Biosafety Level 3 facility as well as transcriptomics studies to assess how macrophages affect the developmental and molecular fate of *Coccidioides* arthroconidia. We determined that murine bone marrow-derived macrophages (BMDMs) stimulated a significant increase in spherule formation and a robust delay in hyphal formation in a phagocytosis-dependent manner. Using RNA-seq to determine the transcriptional response of *Coccidioides* to macrophage co-culture, we observed that the fungus accumulates transcripts associated with *in vitro* spherulation, as well as a unique set of transcripts that were more highly abundant in the presence of macrophages. Together, these studies elucidate how *Coccidioides* adapts to the presence of host immune cells by forming spherules and inducing the expression of potential virulence factors to cause disease.

## RESULTS

### Bone marrow-derived macrophages *promote Coccidioides* spherule development

**Fig 1:**
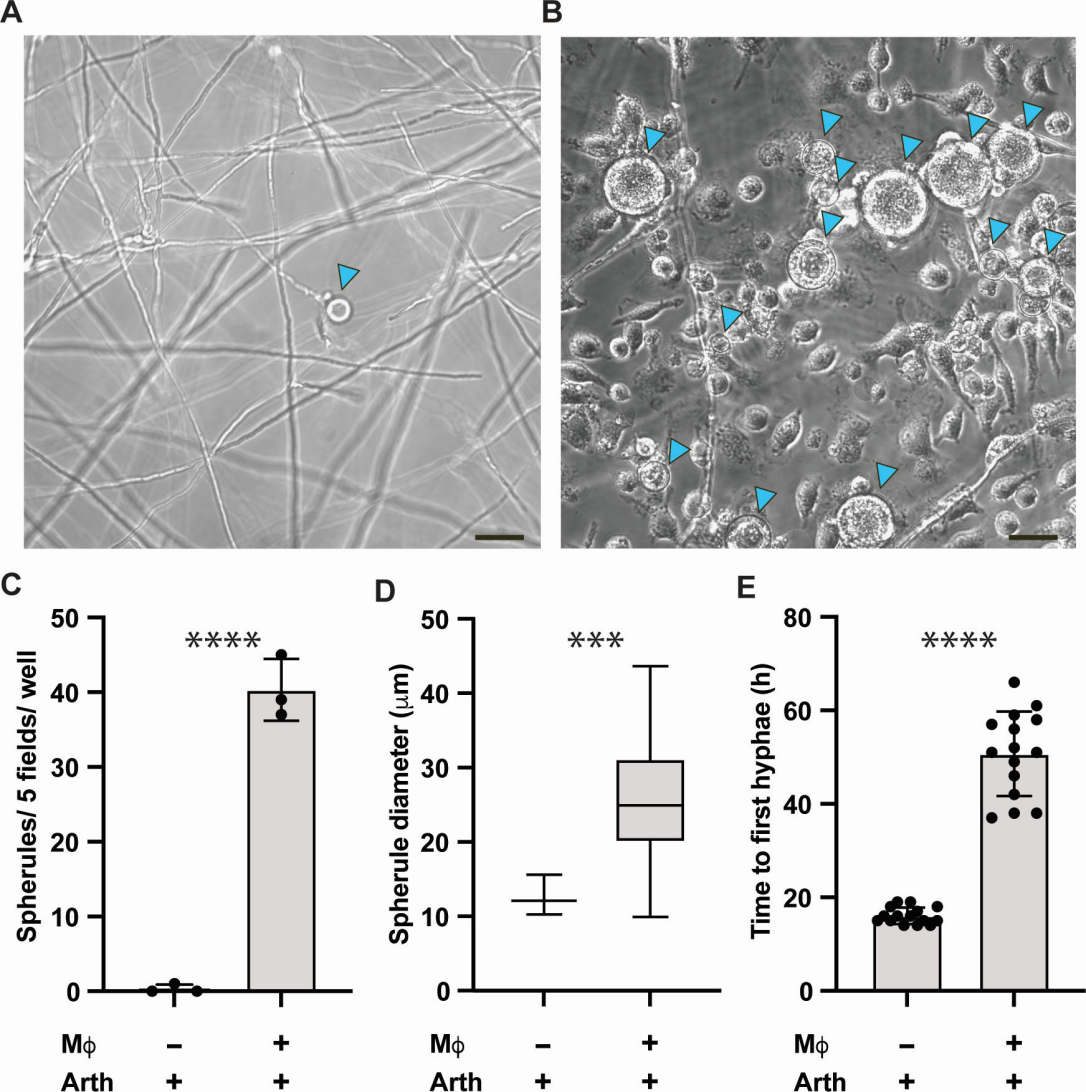
Macrophages promote spherule formation and delay hyphae. Representative image of (**A**) arthroconidia alone or (**B**) arthroconidia with BMDMs at a multiplicity of infection of 0.1 (1 arthroconidia for every 10 BMDMs) on day 3 of the experiment; scale bar represents 30 μM. Representative spherules are labeled with blue arrowheads. (**C**) The number of spherules per well (each point represents data from five fields per well with three independent wells per condition). (**D**) Average diameter of spherules on day 3 of infection in wells with arthroconidia alone or with BMDMs represented as a box and whisker plot. (**E**) Time to first hyphae in frame over 3 days, with pictures taken once an hour, five fields per well. Data presented (C, D, and E) are representative of at least three independent experiments. Unpaired t-test used for panel C and Mann-Whitney test for panels D and E; error bars in panels C and E indicate standard deviation, ***P ≤ 0.001 and ****P ≤ 0.0001.

We used live imaging to compare the germination and subsequent development of arthroconidia in tissue culture conditions (37°C, 5% CO_2_, in bone marrow-derived macrophage media) alone or in the presence of BMDMs. In the absence of macrophages, arthroconidia primarily developed into hyphae, rarely forming spherules (Fig. 1A; Video S1). Strikingly, in the presence of BMDMs, many arthroconidia developed into spherules (Fig. 1B and C; Video S2). The spherules formed in the presence of macrophages were also larger in diameter than spherules formed under tissue culture conditions (Fig. 1D). In addition to promoting spherulation, the presence of macrophages significantly delayed the time it took for hyphae to first be observed in the field, which we described as “time to first hyphae” (Fig. 1E). Since time to first hyphae is a function of both the rates of arthroconidial germination and hyphal growth, we note that either or both could have been affected by BMDMs.

**Fig 2:**
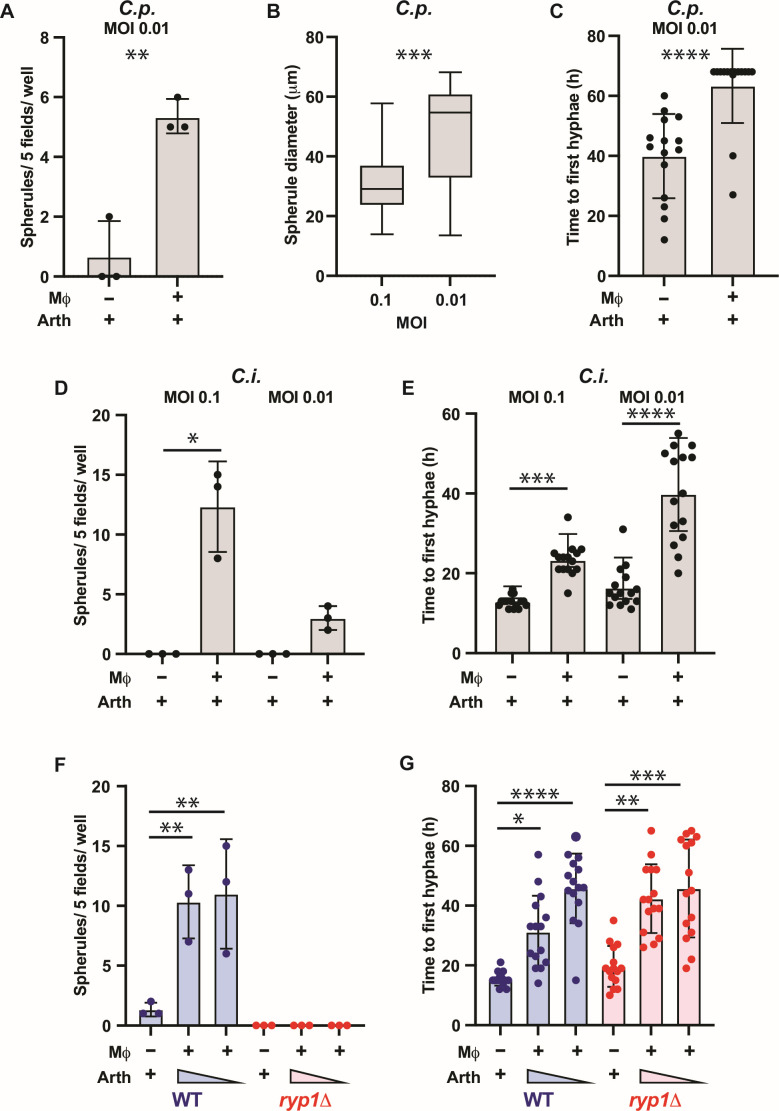
Macrophage induction of spherulation and delay of hyphae is independent of MOI and *Coccidioides* species. (**A**) The number of spherules per well (each point represents data from five fields per well with three independent wells per condition) in wells with arthroconidia alone or BMDMs at MOI 0.01. (**B**) Average diameter of spherules on day 3 of infection at MOI 0.1 or MOI 0.01 represented as a box and whisker plot. (**C**) Time to first hyphae in the frame of arthroconidia grown alone or with BMDMs at MOI 0.01. (**D**) Number of spherules per well and (**E**) time to first hyphae for *Coccidioides immitis* RS arthroconidia alone or with BMDMs at MOI of 0.1 or 0.01. (**F**) Number of spherules per well and (**G**) time to first hyphae of WT and *ryp1∆ C. posadasii* Silveira arthroconidia alone or with BMDMs at MOI 0.1 or 0.05. Pictures taken once an hour, five fields per well. Data for each figure represent five 40× fields counted per well, with three wells per condition. The data presented are representative of at least three independent experiments. Unpaired t-test used for panel A, Mann-Whitney test for panels B and C, one-way ANOVA for panels D and F, and Kruskal-Wallis test for panels E and G, *P ≤ 0.05, **P ≤ 0.01, ***P ≤ 0.001, and ****P ≤ 0.0001.

We investigated whether these morphological findings were affected by the number of arthroconidia per macrophage (multiplicity of infection, or MOI). At the lower MOI of 0.01, there were still significantly more spherules in the presence of macrophages than when the same arthroconidial inoculum was grown alone (Fig. 2A). Notably, the size of the spherules at MOI 0.01 was larger than the spherules that developed at an MOI of 0.1 (average diameter of 54 µm vs. 29 µm) (Fig. 2B). The size of the spherules that formed in the presence of macrophages was more comparable to the size of spherules observed previously *in vivo* (40–100 μm), in contrast to the smaller spherules that are characteristic of *in vitro* spherulation (10–20 μm) (2, 12). At this lower inoculum, the time to observation of first hyphae in the microscopy field was longer, as sparser arthroconidia mean hyphal germination is a rarer event; however, there was still a significant increase in time to first hyphae in the wells with macrophages compared to those without macrophages (Fig. 2C).

These experiments were performed with the *C. posadasii* Silveira strain. To determine if a different *Coccidioides* species behaves similarly in the presence of macrophages, we performed the same experiments with *C. immitis*, which has more than 90% homology in predicted proteins with *C. posadasii* and a similar clinical manifestation (13). We found that macrophages also induced spherulation of *C. immitis* RS arthroconidia (Fig. 2D) and delayed the time to first hyphae (Fig. 2E).

To characterize fungal genes that are required for spherulation in the presence of macrophages, we co-cultured macrophages with mutant arthroconidia lacking the transcription factor *RYP1*. We have previously shown that *RYP1* is essential for the development of the spherule *in vitro* and in a murine infection model (6, 14). We found that *RYP1* is also essential for spherulation in the presence of macrophages (Fig. 2F). Interestingly, we found that the time to first *ryp1Δ* hyphae in the field was delayed in the presence of macrophages (Fig. 2G), implying that macrophages inhibited germination and/or hyphal growth rates of the *ryp1∆* mutant independent of spherule formation.

### Spherule development is dependent on contact with live macrophages

Secreted compounds from macrophages could be responsible for promoting the spherulation of *Coccidioides* during co-culture. To test this hypothesis, we utilized a transwell system to image arthroconidia in the lower well that either had no exposure to macrophages, had macrophages present in the same lower well, or had macrophages seeded on the insert. Macrophages seeded on the insert were separated from *Coccidioides* by a 0.4-μm membrane that allowed media and secreted compounds to move between the insert and main well but prevented direct contact between the arthroconidia and macrophages.

**Fig 3:**
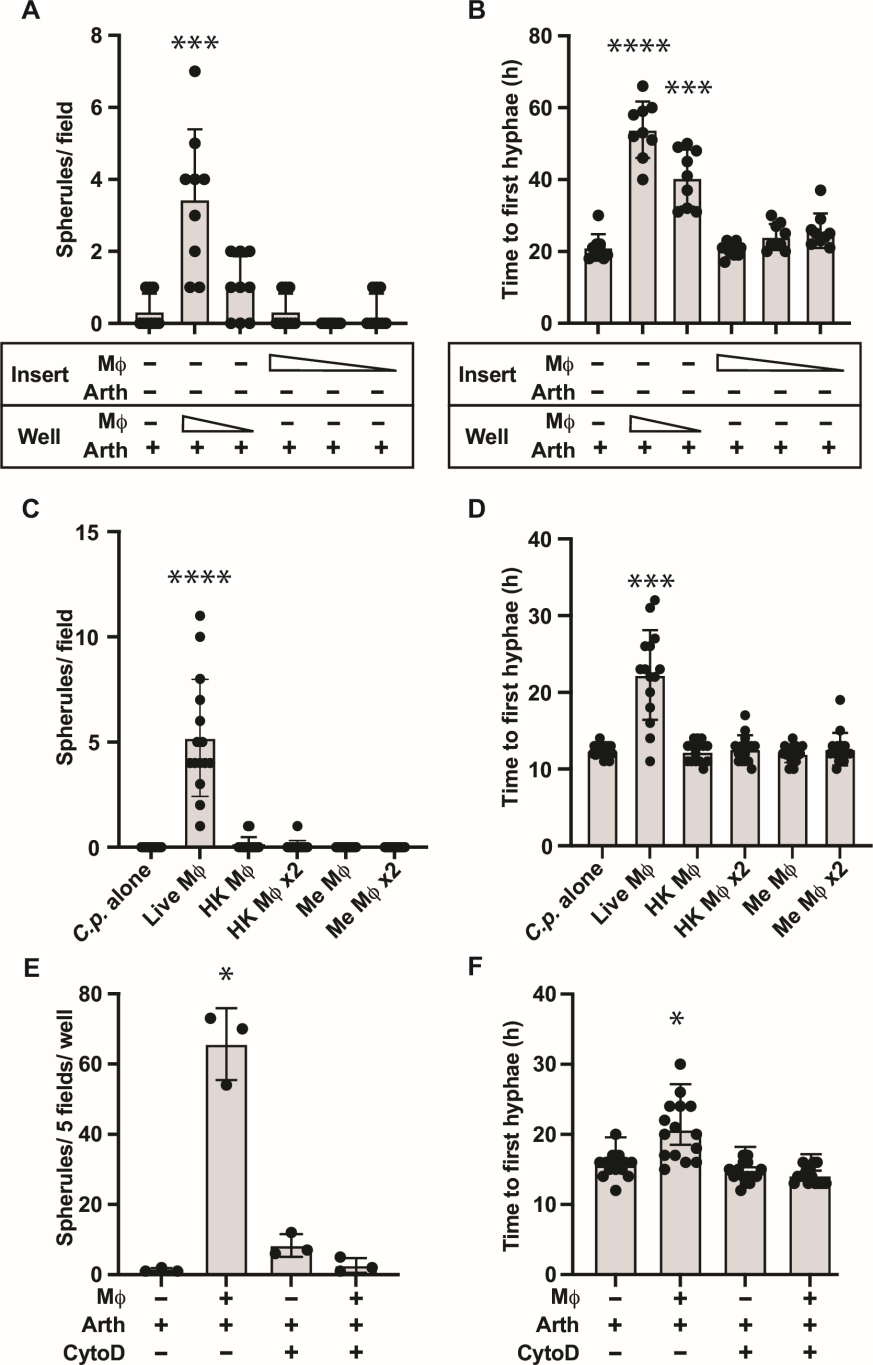
Phagocytosis of arthroconidia is required for macrophages to promote spherule formation and delay hyphae. The number of spherules per field (**A**) and time to first hyphae per field (**B**) for arthroconidia cultured with or without macrophages in the well with arthroconidia or with macrophages separated by a transwell. The number of spherules per well (**C**) and time to first hyphae per field (**D**) arthroconidia with live macrophages or heat-killed or methanol-treated macrophages (at the same density or twice as dense as live macrophage well). The number of spherules per well (**E**) and time to first hyphae per field (**F**) of arthroconidia cultured with or without macrophages in the presence or absence of 10 μM cytochalasin D. For all samples, images were counted on day 3. For panels A and B, nine fields per condition were quantified, and for panels C–F, five fields per well and three wells per condition were quantified. The data presented are representative of at least three independent experiments. Kruskal-Wallis test was used for panels A–D and F, and one-way ANOVA was used for panel E. *P ≤ 0.05, **P ≤ 0.01, ***P ≤ 0.001, and ****P ≤ 0.0001.

The presence of macrophages in the same wells as arthroconidia resulted in an increased number of spherules per field and a delay in time to first hyphae, as expected (Fig. 3A and B). However, when macrophages were separated from arthroconidia by the insert, they were unable to stimulate spherulation (Fig. 3A). Additionally, the time to first hyphae in these wells was indistinguishable from wells with no macrophages (Fig. 3B). These data indicate that contact between arthroconidia and macrophages was essential for spherule stimulation and delay of hyphae.

Since contact with macrophages was necessary for the promotion of spherulation in our conditions, we investigated whether contact with dead macrophages was sufficient to induce spherulation and delay time to first hyphae. We exposed arthroconidia to live macrophages, heat-killed macrophages, or methanol-fixed macrophages. When arthroconidia were plated with BMDMs that were heat-killed or fixed with methanol, there were no changes in spherule number (Fig. 3C) or the time to first hyphae (Fig. 3D). Thus, contact with the surface of dead macrophages was not sufficient to induce spherule development.

### Macrophages do not induce spherulation when phagocytosis is blocked

To test if phagocytosis was required for macrophage promotion of spherulation and delay of hyphae formation, we exposed arthroconidia to BMDMs in the presence of cytochalasin D, an actin polymerization inhibitor that inhibits phagocytosis as well as secretion. There was no increase in spherulation of *Coccidioides* exposed to cytochalasin D-treated macrophages compared to arthroconidia grown alone, with or without cytochalasin D (Fig. 3E). Of note, exposure of arthroconidia to 10 µM cytochalasin D during *in vitro* spherulation conditions did not inhibit the ability to make spherules or produce endospores *in vitro* (Fig. S1). The delay in hyphal formation noted in the presence of macrophages was eliminated if those macrophages were treated with cytochalasin D (Fig. 3F).

### Co-culture with *Coccidioides* arthroconidia leads to macrophage death

Many pathogens that survive phagocytosis by host immune cells manipulate host cell death to survive within or to defend against those cells. We examined the effect of arthroconidia infection on macrophage survival by measuring the release of lactate dehydrogenase (LDH) from host cells as a proxy for host-cell lysis. When macrophages were infected at an MOI of 0.1, there was no significant LDH release until day 2 of infection. When macrophages were infected at a higher MOI of 1, they showed significant LDH release at days 1, 2, and 3 post-infection (Fig. S2). We concluded that co-culture with *Coccidioides* arthroconidia led to the lysis of BMDMs.

### *Coccidioides* induces a unique transcriptional program when challenged with macrophages

To further characterize macrophage-induced spherulation, we performed RNA sequencing of *Coccidioides* co-cultured with macrophages. To overcome the challenge of low fungal RNA yield from co-culture, we utilized multiple MOIs to increase the range of fungal burden at early time points, subjected samples to bead beating to optimize lysis of fungal cells, and increased the sequencing depth.

**Fig 4:**
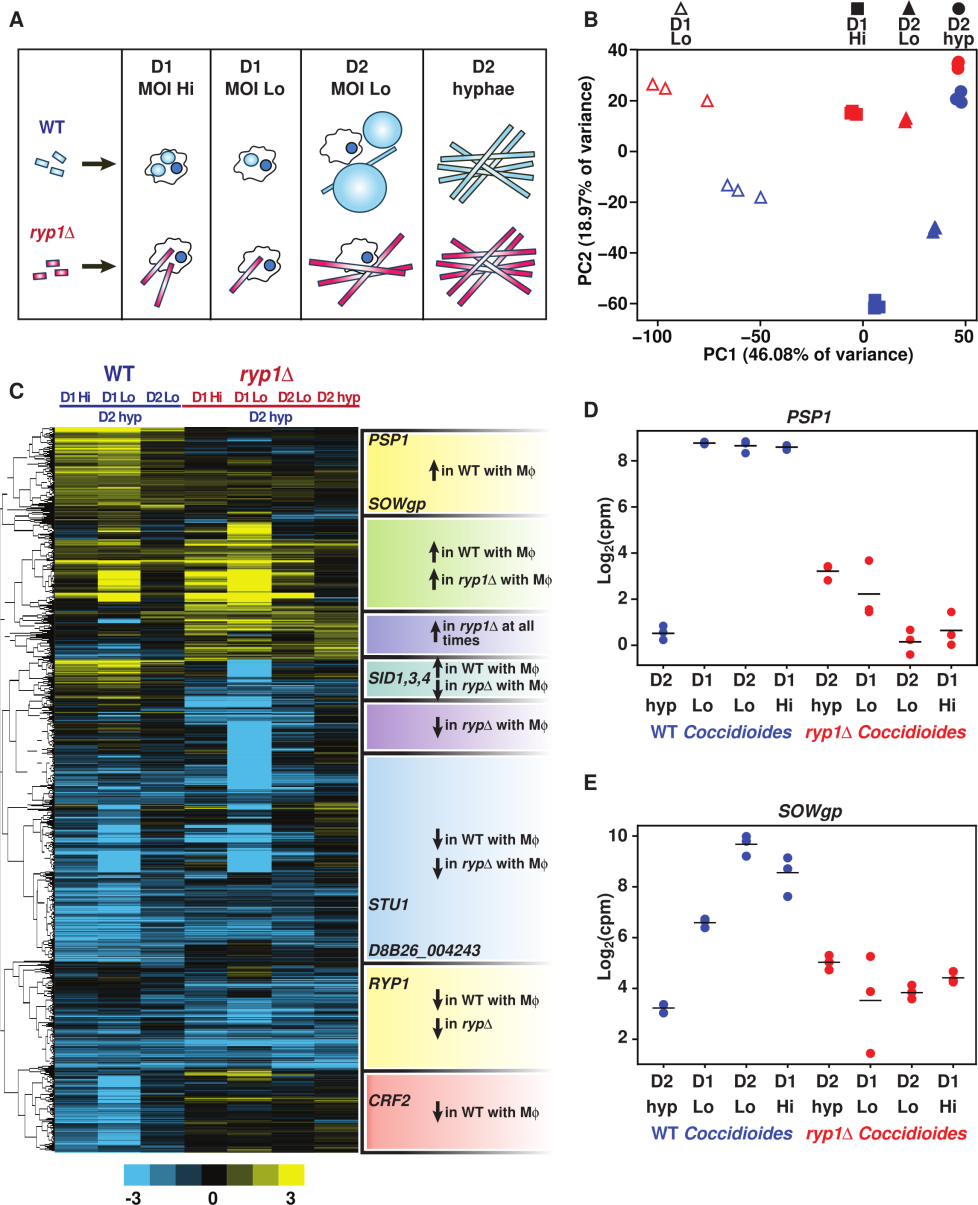
*Coccidioides* induces transcriptome changes in response to BMDMs. (**A**) Schematic of RNA-seq conditions with uninfected macrophages, macrophages exposed to WT arthroconidia (blue), and *ryp1∆* arthroconidia (red). “Hi” indicates MOI = 1, and “Lo” indicates MOI = 0.1. (**B**) PCA plot of each sample with WT in blue, *ryp1∆* in red, *Coccidioides* alone at D2 is represented by circles, *Coccidioides* exposed to BMDMs at Lo MOI by triangles (open at D1 and filled at D2), and Hi MOI at D1 by squares. (**C**) Hierarchically clustered heatmap of differentially expressed transcripts for seven conditions, each compared to WT *Coccidioides* grown alone in tissue culture conditions. The seven conditions are WT or *ryp1∆ Coccidioides* grown in the presence of BMDMs (at the indicated MOI and time point) or *ryp1∆ Coccidioides* grown alone under tissue culture conditions. Heatmap colors indicate log_2_ differential expression ratios relative to WT alone in units of counts per million. Depth-normalized abundance of transcripts (as log_2_ counts per million) of two *Coccidioides* genes known to be expressed in spherules (*PSP1* [**D**] and *SOWgp* [**E**]). Dots represent individual observations, and bars are averages in log space.

We examined the transcriptome of WT *Coccidioides* grown alone for 2 days, or co-cultured with BMDMs at an MOI of 1 (Hi) for 1 day (with greater RNA yield for the early time point) and at an MOI of 0.1 (Lo) for 1 or 2 days (when the majority of macrophages are still viable [Fig. S1]). To distinguish which transcriptome changes may be specific to the formation of spherules rather than the delay of hyphal formation, we leveraged the *ryp1Δ* mutant, which cannot make spherules in the presence of macrophages (Fig. 2F), although it did have a delay in time to first hyphae in the presence of macrophages (Fig. 2G). We reasoned that transcriptome changes that correlated with spherulation would not occur in the *ryp1∆* mutant. Therefore, we profiled the transcriptomes of WT *Coccidioides* and the *ryp1Δ* mutant in the presence of macrophages at the same MOIs and time points, each in triplicate (Fig. 4A).

Transcript abundance was quantified with KALLISTO relative to the most recent *C. posadasii* Silveira gene annotation (GCA_018416015.2). In the text below, we give systematic gene names from this annotation (starting with D8B26_) as well as the corresponding gene names from the previous *C. posadasii* Silveira gene annotation (starting with CPSG_) and orthologous genes from *C. immitis* RS (starting with CIMG_). Further analysis was restricted to 8,274 well-represented transcripts that had at least 10 counts in at least 9 samples (Table S1). Principal component analysis of the expression profiles revealed two independent components accounting for 65% of the variance. The first component accounted for 46% of the variance and separated the samples by exposure to BMDMs and time, regardless of genotype (WT or *ryp1Δ*), and the second component accounted for 19% of the variance and distinguished samples with spherules from those with majority hyphae (i.e., WT *Coccidioides* with BMDMs vs. WT alone or any of the *ryp1Δ* samples) (Fig. 4B).

Differential expression analysis was carried out with the negative binomial model of edgeR to account for counting noise in the low-depth data (due to limiting fungal material in co-culture), combined with log-normally distributed biological and replicate variance. For the 8,274 well-sampled transcripts, we estimated contrasts for each WT and *ryp1Δ* infection time point, and for *ryp1Δ* alone, each relative to WT alone; 3,753 transcripts were at least 2-fold differential in at least one of these seven contrasts at a false discovery rate (FDR) of 5% (Fig. 4C; Table S2). Hierarchical clustering revealed eight distinct expression patterns as indicated by the colored regions on the right side of the heat map (Fig. 4C). More transcripts decreased in abundance in response to macrophages in both WT and *ryp1Δ Coccidioides* compared to WT arthroconidia grown in the same conditions in the absence of host cells (“D2 hyp”). Interestingly, prior reports of *in vitro* grown young spherules vs. mature hyphal cultures also noted that more genes were downregulated than upregulated in early spherules compared to mature hyphae (15).

Approximately one-third of the differentially expressed transcripts showed increased abundance in WT *Coccidioides* in response to BMDMs, including genes previously identified as encoding spherule-associated factors, such as *SOWgp*, *PSP1*, *MDR1*, and *OPS1* (Fig. 4C, D, and E) (16, 17). Other transcripts included in this subset were *PDC1* (putative pyruvate decarboxylase), *UAZ1* (putative urate oxidase), and *CCC1* (a putative calcium transporter). BMDM-induced genes in WT *Coccidioides* also included transcripts encoding hypothetical proteins of interest that were previously shown to be more significantly induced in animal models vs. *in vitro,* including D8B26_007421 (CPSG_01366/CIMG_09001, a putative secreted protein), D8B26_000085 (CPSG_05795/CIMG_05576, an immunoreactive protein that has been found in sera from immunized or *Coccidioides*-infected dogs) (18), and D8B26_005342 (CIMG_00509, another putative secreted protein) (19). Thus, our data suggested that the presence of macrophages could sufficiently mimic the host environment to elicit the transcription of these host-associated genes in *Coccidioides*.

We were interested in transcripts that were dependent on *RYP1* for increased abundance since these transcripts might correlate with the ability of the fungus to generate spherules. Transcripts that were increased in WT *Coccidioides* exposed to BMDMs but lower in *ryp1Δ Coccidioides* exposed to BMDMs included D8B26_001957 (encoding a protein containing a Major Facilitator Superfamily [MFS] domain), D8B26_005576 (ortholog of iron transport multicopper oxidase *FET3*), and D8B26_000555 (a urease). Members of the siderophore biosynthesis cluster (genes *SID3*, *SID4*, *NPS1*, *OXR1*, and *MFS1* [20, 21]) were also in this group, but interestingly, they were only significantly increased in abundance in the WT samples exposed to BMDMs at day 1 but not at day 2. This temporal regulation could be due to the local environment—at day 1, many of the WT *Coccidioides* remained within or closely associated with macrophages (potentially experiencing iron deprivation), whereas by day 2, the spherules were larger and no longer internalized.

Macrophage-induced transcripts common to both WT and *ryp1Δ Coccidioides* could represent a generalized *Coccidioides* response to the stress of host cells independent of spherulation. One example was D8B26_008184, which exhibits homology to the transcription factors RlmA from *Aspergillus niger* and Smp1/Rlm1 from *Saccharomyces cerevisiae* and is proposed to be induced by cell wall stress in other fungi (22–24). Another gene with increased transcript abundance in all samples with BMDMs was D8B26_001686, which encodes a protein of the Nramp family of metal transport proteins (similar to *S. cerevisiae* Smf1-3 transporters), which suggests that phagocytosed arthroconidia experience differences in metal availability within the host cell. D8B26_002597, which was enriched in all samples with BMDMs, especially at 24 h, appears to encode a non-ribosomal peptide synthase-like protein that might generate a natural product in response to host cells. These changes in the transcriptome in response to macrophages highlight that the arthroconidia are likely experiencing cell wall stress and changes in nutrient availability and thus induce the transcription of a set of genes independent of spherulation.

Since BMDMs promoted *Coccidioides* spherulation and inhibited hyphal growth, we expected that hyphal-associated transcripts would show decreased abundance in WT *Coccidioides* exposed to BMDMs. Indeed, many genes associated with hyphal growth in *Coccidioides* and other fungal pathogens were downregulated in WT *Coccidioides* during macrophage infection. One such gene was D8B26_004243, which encodes a putative Zn_2_C_6_ transcription factor orthologous to *Histoplasma* Nos1, which is upregulated in hyphae, as well as *Aspergillus* spp. RosA and NosA, a putative regulator of sexual development (25, 26). Transcript levels of D8B26_003897, a putative glucosidase similar to the *A. fumigatus* hyphal-associated glycosylhydrolase Crf2 (27), also showed decreased abundance in WT *Coccidioides* in the presence of macrophages. The *Coccidioides* orthologs of two transcription factors that are key players in the hyphal program in *Histoplasma*, Stu1 (D8B26_002234), and Fbc1 (D8B26_003963) (28, 29), both showed decreased transcript abundance in WT *Coccidioides* in the presence of macrophages. In the case of the *ryp1* mutant, although there was also a delay in the appearance of hyphae during macrophage co-culture (Fig. 2G), the mutant is unable to form spherules and can only grow as hyphae. Correspondingly, for the majority of hyphal-associated genes, there was no obvious decrease in expression at the time points we surveyed.

### *Coccidioides* transcripts induced during macrophage co-culture include both core and host-specific spherule-associated genes

**Fig 5:**
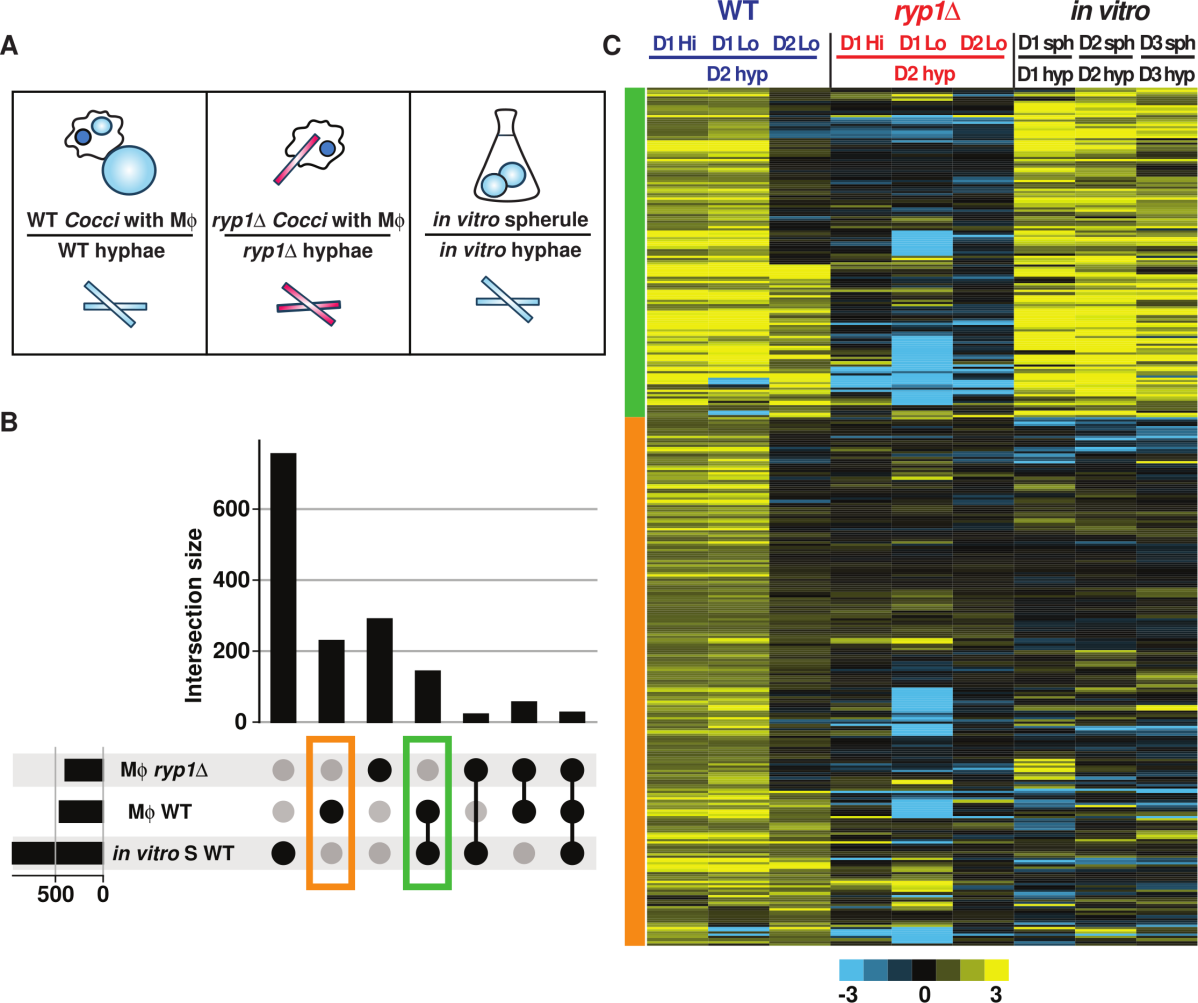
BMDMs trigger the induction of core spherule-associated genes and macrophage-specific spherule-associated genes. (**A**) Schematic of different samples used for the analysis in panel B. (**B**) UpSet plot comparing the different gene sets depicted in panel A. “Mɸ WT” are transcripts with significantly increased abundance in at least two-thirds of the WT *Coccidioides* samples exposed to BMDMs vs. WT *Coccidioides* alone; “Mɸ *ryp1*” are transcripts with significantly increased abundance in at least two-thirds of the *ryp1∆ Coccidioides* samples exposed to BMDMs compared to *ryp1∆ Coccidioides* alone, and “*in vitro* S WT” are transcripts with significantly increased abundance in at least two-thirds *in vitro* spherule samples compared to *in vitro* hyphae at the same time point (6). The horizontal black bars on the left show the size of each individual set of transcripts with increased abundance as described above. The vertical black bars show the magnitude of the overlap between each of these sets (overlapping sets are indicated by connected black circles on the bottom). Each gene can only be assigned to one category per condition. The green box surrounds the subset of transcripts with significantly increased abundance in both *in vitro* and BMDM-associated spherules. Orange box surrounds subset of transcripts with significantly increased abundance only in WT *Coccidioides* exposed to BMDMs. (**C**) Heatmap of transcript abundance for WT *Coccidioides* grown in the presence of BMDMs at different time points and MOIs vs. WT *Coccidioides* grown alone at day 2 (D2 hyp); *ryp1∆ Coccidioides* grown in the presence of BMDMs at different time points and MOIs vs. *ryp1∆ Coccidioides* grown alone at day 2 (D2 hyp); or WT *Coccidioides* grown *in vitro* as spherules at 39°C at 10% CO_2_ at individual time points compared to hyphae grown at 25°C at the corresponding time points. The green and orange bars indicate the same sets of transcripts as marked in panel B. Heatmap colors indicate log_2_ differential expression ratios in units of counts per million.

We hypothesized that the presence of BMDMs induced a core set of spherule-associated genes that were also induced under *in vitro* spherulation conditions, as well as a set of potential virulence factors or effectors that were specific to the presence of host cells. To identify the core genes, we leveraged our laboratory’s recent work creating a transcriptomic atlas of *Coccidioides* arthroconidia, developing spherules, and developing hyphae (6). We compared the gene expression data from the following three sets (Fig. 5A; Table S3): (i) WT *Coccidioides* with BMDMs (spherule) vs. WT *Coccidioides* alone (hyphal); (ii) *ryp1∆ Coccidioides* with BMDMs (hyphal) vs. *ryp1∆ Coccidioides* alone (hyphal); and (iii) *in vitro* spherules vs. *in vitro* hyphae at days 1, 2, or 3 post-germination. We considered genes in a given set to be induced if their transcripts were significantly more abundant for at least two of the three conditions in that set. *In vitro* spherules had 947 differential transcripts with increased abundance in comparison to *in vitro* hyphae, the majority of which were not shared with the other contrasts, possibly due to the unique temperature and CO_2_ utilized to culture these cells *in vitro*. In the presence of BMDMs, WT and *ryp1Δ Coccidioides* had 455 and 395 differential transcripts with increased abundance compared to each of their corresponding *Coccidioides* alone controls. Although a majority of the differentially expressed transcripts in response to macrophages were unique to either WT or *ryp1Δ Coccidioides*, 56 were shared only among the two. Interestingly, the shared set could represent stress-response transcripts that increased in abundance during macrophage co-culture independent of spherule formation and Ryp1 activity. More significantly, this analysis indicates that the majority of genes induced in wild-type *Coccidioides* in the presence of macrophages were dependent on Ryp1 and/or spherule formation.

We then identified a set of 143 core spherule-enriched transcripts with increased abundance in both WT *Coccidioides* in the presence of macrophages and *in vitro* spherules (green box in Fig. 5B and green bar in Fig. 5C; Table S4). As proof of principle, we note this set included many of the previously identified spherule-associated genes such as *SOWgp*, *PSP1*, *OPS1*, *PDC1*, *UAZ1*, and *TYR2* (16, 17). The 229 transcripts that showed higher abundance only in WT *Coccidioides* in the presence of macrophages (orange box in Fig. 5B and orange bar in Fig. 5C; Table S5) could represent virulence factors or other proteins required by *Coccidioides* during interaction with macrophages.

During this analysis, we noted that not all transcripts showed a sustained increase in expression throughout the experiment. To further analyze the expression patterns, we examined the temporal and MOI-dependent expression of the core spherule-specific genes (Fig. 5C, upper/green bar) and macrophage-induced genes (Fig. 5C, lower/orange bar). The final subsets were independently hierarchically clustered and concatenated into a single heatmap (Fig. 5C). Half of the core genes were up in all three WT-with-BMDM conditions, with the other half up only in the day 1 samples. For the macrophage-induced genes, 70% were up only in the day 1 samples and not in the day 2 samples. This observation is intriguing because, in the context of macrophage co-culture, the developing spherules tended to be intracellular at day 1 and extracellular at day 2, suggesting that these fungal cells were in distinct environments. One of the most interesting transcripts with increased abundance in WT *Coccidioides* associated with BMDMs was D8B26_000085, which encodes a highly seroreactive protein that is found in infected human lungs (19). Antibodies to D8B26_000085 persist in the serum of naturally infected or vaccinated dogs (18), indicating that it is expressed and antigenic in animal infections. Other genes with similar patterns included D8B26_005548 (which contains an MFS domain), D8B26_000635 (a cytochrome c peroxidase), and D8B26_006970 (a presumed amidase).

To focus on potential secreted effectors that were induced when *Coccidioides* was co-cultured with macrophages, we determined which transcripts were predicted to encode proteins with a signal peptide; 48 transcripts met this criterion, including *SOWgp*, *TYR2*, and D8B26_000085 (CPSG_05795/CIMG_05576). Next, since many fungal effectors in plant pathogens (30) and in *Histoplasma* (31) are small, cysteine-rich proteins, we further narrowed the list to small proteins that were cysteine-rich (<250 amino acids and ≥4 cysteines); 10 genes encoded proteins that fit these criteria, including the superoxide dismutase *SOD3* (D8B26_006671), which is a virulence factor in *Histoplasma* (32); *ELI1* (D8B26_007114), which is a protective antigen of unknown function (33); two small hypothetical proteins previously shown to be abundant *in vivo* (D8B26_007421 [CPSG_01366] and D8B26_005342 [CIMG_00509]); a predicted cutinase, which is expressed in the WT day 1 samples with BMDMs (D8B26_005437); and an ortholog of *A. nidulans* CalA that plays a role in conidial germination (D8B26_001315) (34). These transcripts may represent key molecules at the interface between the host and pathogen during *Coccidioides* infection.

## DISCUSSION

As the endemic region for *Coccidioides* expands, putting more people at risk of disease, there is an urgent need for fundamental knowledge of how this pathogen responds to the host to cause disease. Here, we showed that phagocytosis of *Coccidioides* arthroconidia by macrophages induced development of the pathogenic form of *Coccidioides*, allowing the fungus to subvert these key innate immune cells to cause disease. Furthermore, we show that *Coccidioides* exposed to BMDMs expressed key genes for spherule growth as well as potential virulence factors. Finally, we have identified a core set of spherule-associated transcripts with higher abundance in spherules, independent of temperature and CO_2_ conditions. This set will be critical for identifying targets for diagnostics or vaccine candidates in the future.

One distinguishing feature of *Coccidioides* in comparison to many other fungal pathogens is its ability to cause disease in immunocompetent individuals. The ability of *Coccidioides* arthroconidia to respond to macrophages by inducing spherule development and the subsequent difficulty of phagocytes to destroy the large spherule may contribute to the ability of this environmental fungus to defy the innate immune response. We have shown that after phagocytosis by macrophages, arthroconidia respond by forming spherules and that the average size of macrophage-associated spherules is larger than observed during *in vitro* spherule development. *Coccidioides* exposure resulted in macrophage cell lysis, suggesting that intracellular *Coccidioides* might cause a physical strain that provokes host cell death or that *Coccidioides* might actively trigger a host cell-death pathway to escape the macrophage. Alternatively, macrophage lysis might be an effort on the part of the host cell to injure the pathogen and limit infection or stimulate immune cell recruitment. Fungi and other pathogens have developed strategies to manipulate host cell death pathways to their advantage (35–38), and investigating this process in *Coccidioides* infections may elucidate key elements of pathogenesis. This interaction between the fungus and innate immune cells could contribute to disease progression and potentially to the dissemination of the organism to extra-pulmonary sites. Notably, the specifics of the host response, which are likely influenced by the initial interaction of the fungus with phagocytes, are likely to play a role in dissemination since it is known that polymorphisms in IFNγ, IL-12, and Dectin-1 are associated with the likelihood of disseminated coccidioidomycosis (39).

This work represents the first transcriptional study of *Coccidioides* in the presence of macrophages. We overcame the technical challenges of a relatively low *Coccidioides* RNA to macrophage RNA ratio with efficient disruption of fungal cells, choice of time points, and multiplicity of infection, as well as increased depth of sequencing. One of the strengths of this study is that the conditions where WT *Coccidioides* grew as hyphae or as spherules had identical CO_2_ levels and temperature; thus, differential genes in the data set reflect factors associated with spherule development and host-pathogen environment rather than temperature or elevated CO_2_ alone. The identification of a core set of spherule-associated genes will be useful to our understanding of spherule biology as well as the development of *Coccidioides* vaccines. Additionally, we were able to observe nuance in the timing of gene expression. *Coccidioides* transcripts that are only expressed early in macrophage co-culture may reflect a germination program that gives rise to spherules, such as D8B26_001315, the ortholog of *A. nidulans* CalA. D8B26_001315 is up in early WT *Coccidioides* samples exposed to BMDMs as well as in early *in vitro* spherules. Additionally, it was striking that macrophages induced the increased abundance of transcripts encoding *Coccidioides* proteins that were previously observed in human lung samples, including D8B26_000085, one of the most immunogenic proteins encoded by *Coccidioides*. These observations suggest that macrophage-*Coccidioides* co-culture provides a simplified model system for analyzing important host-fungal interactions.

An open question is the mechanism used by arthroconidia to sense the intracellular environment to induce the spherule form. The phagosome is a complex and dynamic environment that poses many potential challenges to enclosed pathogens, including changes in pH, exposure to reactive oxygen species, and limitation of nutrients. What role each of those processes plays in spherulation or inhibition of hyphal germination is unknown. Furthermore, spherule formation ultimately results in the release of internal cells called endospores, and the interaction of macrophages and endospores remains to be fully explored. An understanding of how these different *Coccidioides* cell types subvert the host immune response will provide strategies to leverage or modulate the host immune response to promote disease resolution instead of progression.

## MATERIALS AND METHODS

### Strains

Most experiments were conducted with the WT *C. posadasii* strain Silveira (BEI # NR-48944), a kind gift from Dr. Bridget Barker, Northern Arizona University. We also utilized *C. immitis* strain RS (BEI # NR-48942) and *ryp1Δ* deletion mutant in the *C. posadasii* strain Silveira background (6, 14).

### Cell culture

Bone marrow-derived macrophages (BMDMs) were derived from bone marrow obtained from the femurs and tibias of 6- to 8-week-old female C57BL/6 mice as previously described (40). Bone marrow cells were grown in bone marrow macrophage media (BMM) containing Dulbecco’s modified Eagle medium (DMEM high glucose) with 10% conditioned media from CMG 14-12 cells (source of m-CSF), 20% fetal bovine serum (FBS), 110 µg/mL penicillin and streptomycin, 2 mM L-glutamine, and 110 µg/mL sodium-pyruvate, for 7 days then frozen in 40% FBS and 10% DMSO for future use. For each experiment, an aliquot was thawed and plated to rest overnight before each experiment. Cultures were maintained at 37°C and 5% CO_2_.

### Live imaging

Live imaging was performed on the Etaluma LS720 microscope with Etaluma camera with Aptina sensor using the Olympus phase contrast 40× objective with air lens (LCACHN40XIPC). Cells were maintained in a stage top incubator (Okolab) to control temperature, humidity, and CO_2_. For most time course experiments, brightfield images of 5–9 fields per well were taken once an hour. The number of spherules per field was quantified at the final time point. Spherule number per well was defined as the aggregate count of spherules in five fields per well. Spherule diameter was measured for all spherules in the field using Fiji (ImageJ2 version: 2.9.0). Time to first hyphae was quantified as the time when the first arthroconidia germinated into a hypha in the field of view or a hypha entered the field of view.

Unless otherwise noted, experiments were performed with BMDMs in 48-well tissue culture-treated cell culture plates (Corning 3548), seeded at 0.75–1 × 10^5^ macrophages per well and infected at an MOI of 0.1 arthroconidia per macrophage. Other MOIs utilized were 0.01 and 1. For transwell experiments, six-well transwell plates (Costar 3450) were seeded with macrophages at varying densities in the well or in the transwell.

For the experiments with dead macrophages, BMDMs were heat-killed at 65°C for 5 min or methanol-fixed by resuspending in 70% methanol for 10 min, then spun for 5 min at 1,200 rpm and resuspended in fresh BMM before plating. Cells were plated at the same density as the live BMDMs (1 × 10^5^ cells per well) or at twice the density (2 × 10^5^ cells per well).

Cytochalasin D (Sigma) was resuspended in DMSO and added at a final concentration of 10 μM to BMDM 30 min prior to the addition of arthroconidia and remained throughout the infection. Arthroconidia were grown in converse media with 10 µM cytochalasin D or DMSO in spherulation conditions (shaking culture at 39°C, 10% CO_2_) (5).

### Lactate dehydrogenase (LDH) release assay

BMDMs were seeded (7.5 × 10^4^ cells per well) in 48-well plates and infected at an MOI of 0.1 or 1 in triplicate as described above. At the indicated time points, supernatants were removed from triplicate wells and spun at 550 × *g* to pellet debris. The resulting supernatants were stored at 4°C until LDH quantification, as described previously (41). BMDM lysis was calculated as the percentage of total LDH from supernatant of wells of uninfected macrophages lysed in 1% Triton X-100 at the time of infection. The total LDH at later time points can be greater than the total LDH from the initial time point due to continued replication of BMDMs over the course of the experiment, resulting in an apparent lysis that is greater than 100%.

### RNA-seq analysis

BMDMs were seeded 1 day prior to the infection in 6-well plates. On the day of infection, WT and *ryp1Δ* arthroconidia were added to the wells at an MOI of 0.1 or 1, and the plates were spun at 550 × *g* for 5 min. After 1 h, the media were removed and replaced with fresh BMM, except for the *Coccidioides* alone wells. At each time point, the cells were washed twice with PBS, and then QIAzol (Qiagen) was added to the wells. After 5 min, duplicate wells were then combined for each sample, with triplicate samples collected for each condition. Each sample underwent bead beating for 2 min with 0.5 mm zirconia/silica beads (BioSpec) to fully lyse fungal cells prior to RNA extraction.

RNA was extracted using the Direct-zol RNA Miniprep Plus isolation kit (Zymo). Libraries were made with the NEBNext polyA mRNA magnetic isolation module and NEBNext Ultra II Directional RNA Library Prep kit with dual-indexed multiplexing barcodes (New England Biolabs) per the manufacturer’s instructions. Libraries were pooled, and sequencing was performed by the Chan-Zuckerberg BioHub-San Francisco on one lane of Novaseq S4 (Illumina).

Transcript abundances were quantified based on the annotated *C. posadasii* Silveira genome (42), BioProject PRJNA664774. Relative abundances (reported as TPM values [43]) and estimated counts (est_counts) of each transcript in each sample were estimated by alignment-free comparison of k-mers between the reads and mRNA sequences using KALLISTO version 0.46.2 (44). Further analysis was restricted to transcripts with estimated counts ≥10 in at least nine samples.

Differentially expressed genes were identified by comparing replicates for contrasts of interest using the glmQLFit, glmQLFTest, and topTags functions in edgeR version 4.0.16 (45). Genes were considered significantly differentially expressed if they were statistically significant (at 5% FDR) with an effect size of at least 2× (absolute log2 fold change ≥1) for a given contrast. We estimated contrasts for each of the WT and *ryp1∆* infection conditions, Hi MOI (1) day 1 and Lo MOI (0.1) days 1 and 2, as well as *ryp1∆* alone, all relative to WT alone, which grew as hyphae (D2 hyp). Transcripts were clustered on these seven contrasts using maximum linkage hierarchical clustering with uncentered Pearson distances as implemented in Bio.Cluster in BIOPYTHON 1.83 (46).

To compare macrophage-induced gene expression changes to those induced by *in vitro* spherulation conditions, we utilized data from Homer et al., 2025 (6). For the *in vitro* comparisons, we contrasted arthroconidia grown in spherulation conditions (39°C, 10% CO_2_, in Converse media, shaking) and *in vitro* hyphae conditions (30°C, ambient CO_2_, in Converse media, shaking) on days 1, 2, and 3. For the comparisons with macrophages, we contrasted the gene expression of our WT and *ryp1Δ Coccidioides* in the presence of BMDMs (Hi MOI D1, Lo MOI D1, and Lo MOI D2) to the same strain grown alone at day 2 in the hyphal form (D2 hyp). Genes were considered induced in each set if they were significantly different for at least two of the three conditions in that set.

## Data Availability

RNA-seq data are deposited in GEO under the accession number GSE311135.

## SUPPLEMENTAL MATERIAL

Supplemental figures mbio.00492-26-s0001.pdf Figures S1 and S2.

Supplemental Legends mbio.00492-26-s0002.docx Legends for supplemental videos, figures, and tables.

Table S1 mbio.00492-26-s0003.txt Transcript abundances and differential expression of *Coccidioides* grown alone or in the presence of BMDMs.

Table S2 mbio.00492-26-s0004.txt Transcripts differentially expressed in at least one condition.

Table S3 mbio.00492-26-s0005.txt Comparative profiles of *Coccidioides* germinating in the presence of macrophages or *in vitro*.

Table S4 mbio.00492-26-s0006.txt Core spherule-enriched transcripts.

Table S5 mbio.00492-26-s0007.txt Macrophage-specific spherule-enriched transcripts.

Table S6 mbio.00492-26-s0008.txt Candidate secreted effectors.

Video S1 mbio.00492-26-s0009.avi *Coccidioides* arthroconidia grow primarily as hyphae in tissue culture conditions.

Video S2 mbio.00492-26-s0010.avi *Coccidioides* arthroconidia grown in the presence of macrophages develop into spherules.

ASM does not own the copyrights to Supplemental Material that may be linked to, or accessed through, an article. The authors have granted ASM a non-exclusive, world-wide license to publish the Supplemental Material files. Please contact the corresponding author directly for reuse.

## References

[1] Galgiani JN, Ampel NM, Blair JE, Catanzaro A, Geertsma F, Hoover SE, Johnson RH, Kusne S, Lisse J, MacDonald JD, Meyerson SL, Raksin PB, Siever J, Stevens DA, Sunenshine R, Theodore N. 2016. 2016 Infectious diseases society of America (IDSA) clinical practice guideline for the treatment of coccidioidomycosis. Clin Infect Dis 63:e112–46. doi:10.1093/cid/ciw360 27470238

[2] Cole GT, Hung CY. 2001. The parasitic cell wall of Coccidioides immitis. Med Mycol 39:31–40. doi:10.1080/mmy.39.1.31.40 11800267

[3] Lones GW, Peacock CL. 1960. Role of carbon dioxide in the dimorphism of Coccidioides immitis. J Bacteriol 79:308–309. doi:10.1128/jb.79.2.308-309.1960 14418103 PMC278679

[4] Converse JL. 1956. Effect of physico-chemical environment on spherulation of Coccidioides immitis in a chemically defined medium. J Bacteriol 72:784–792. doi:10.1128/jb.72.6.784-792.1956 13398364 PMC358002

[5] Homer CM, Ochoa E, Voorhies M, Sil A. 2024. Optimizing in vitro spherulation cues in the fungal pathogen Coccidioides. mSphere 10:e00679-24. doi:10.1128/msphere.00679-24 39688406 PMC11774042

[6] Homer CM, Voorhies M, Walcott K, Ochoa E, Sil A. 2025. Transcriptomic atlas throughout Coccidioides development reveals key phase-enriched transcripts of this important fungal pathogen. PLoS Biol 23:e3003066. doi:10.1371/journal.pbio.3003066 40233121 PMC12077801

[7] Garfoot AL, Rappleye CA. 2016. Histoplasma capsulatum surmounts obstacles to intracellular pathogenesis. FEBS J 283:619–633. doi:10.1111/febs.13389 26235362 PMC4827932

[8] Chandra P, Grigsby SJ, Philips JA. 2022. Immune evasion and provocation by Mycobacterium tuberculosis. Nat Rev Microbiol 20:750–766. doi:10.1038/s41579-022-00763-4 35879556 PMC9310001

[9] Galgiani JN, Hayden R, Payne CM. 1982. Leukocyte effects on the dimorphism of Coccidioides immitis. J Infect Dis 146:56–63. doi:10.1093/infdis/146.1.56 7086205

[10] Beaman L. 1987. Fungicidal activation of murine macrophages by recombinant gamma interferon. Infect Immun 55:2951–2955. doi:10.1128/iai.55.12.2951-2955.1987 3119493 PMC260012

[11] Baker O, Braude AI. 1956. A study of stimuli leading to the production of spherules in coccidioidomycosis. J Lab Clin Med 47:169–181. 13295674

[12] Mead HL, Teixeira M de M, Galgiani JN, Barker BM. 2019. Characterizing in vitro spherule morphogenesis of multiple strains of both species of Coccidioides. Med Mycol 57:478–488. doi:10.1093/mmy/myy049 30053114 PMC6506604

[13] Sharpton TJ, Stajich JE, Rounsley SD, Gardner MJ, Wortman JR, Jordar VS, Maiti R, Kodira CD, Neafsey DE, Zeng Q, Hung C-Y, McMahan C, Muszewska A, Grynberg M, Mandel MA, Kellner EM, Barker BM, Galgiani JN, Orbach MJ, Kirkland TN, Cole GT, Henn MR, Birren BW, Taylor JW. 2009. Comparative genomic analyses of the human fungal pathogens Coccidioides and their relatives. Genome Res 19:1722–1731. doi:10.1101/gr.087551.108 19717792 PMC2765278

[14] Mandel MA, Beyhan S, Voorhies M, Shubitz LF, Galgiani JN, Orbach MJ, Sil A. 2022. The WOPR family protein Ryp1 is a key regulator of gene expression, development, and virulence in the thermally dimorphic fungal pathogen Coccidioides posadasii. PLoS Pathog 18:e1009832. doi:10.1371/journal.ppat.1009832 35385558 PMC9015156

[15] Carlin AF, Beyhan S, Peña JF, Stajich JE, Viriyakosol S, Fierer J, Kirkland TN. 2021. Transcriptional analysis of Coccidioides immitis mycelia and spherules by RNA sequencing. J Fungi (Basel) 7:366. doi:10.3390/jof7050366 34067070 PMC8150946

[16] Delgado N, Hung CY, Tarcha E, Gardner MJ, Cole GT. 2004. Profiling gene expression in Coccidioides posadasii. Med Mycol 42:59–71. doi:10.1080/1369378031000156890 14982115

[17] Hung CY, Yu JJ, Seshan KR, Reichard U, Cole GT. 2002. A parasitic phase-specific adhesin of Coccidioides immitis contributes to the virulence of this respiratory Fungal pathogen. Infect Immun 70:3443–3456. doi:10.1128/IAI.70.7.3443-3456.2002 12065484 PMC128074

[18] Koehler MA, Song L, Grill FJ, Shubitz LF, Powell DA, Galgiani JN, Orbach MJ, Robb EJ, Chung Y, Williams SA, Murugan V, Park JG, LaBaer J, Lake DF, Magee DM. 2024. Discovery of a unique set of dog-seroreactive Coccidioides proteins using nucleic acid programmable protein array. J Fungi (Basel) 10:307. doi:10.3390/jof10050307 38786662 PMC11121964

[19] Mitchell NM, Dasari S, Grys TE, Lake DF. 2020. Laser capture microdissection-assisted protein biomarker discovery from Coccidioides-infected lung tissue. J Fungi (Basel) 6:365. doi:10.3390/jof6040365 33327604 PMC7765061

[20] Hwang LH, Mayfield JA, Rine J, Sil A. 2008. Histoplasma requires SID1, a member of an iron-regulated siderophore gene cluster, for host colonization. PLoS Pathog 4:e1000044. doi:10.1371/journal.ppat.1000044 18404210 PMC2275787

[21] Hwang LH, Seth E, Gilmore SA, Sil A. 2012. SRE1 regulates iron-dependent and -independent pathways in the fungal pathogen Histoplasma capsulatum. Eukaryot Cell 11:16–25. doi:10.1128/EC.05274-11 22117028 PMC3255934

[22] Damveld RA, Arentshorst M, Franken A, vanKuyk PA, Klis FM, van den Hondel CAMJJ, Ram AFJ. 2005. The Aspergillus niger MADS-box transcription factor RlmA is required for cell wall reinforcement in response to cell wall stress. Mol Microbiol 58:305–319. doi:10.1111/j.1365-2958.2005.04827.x 16164567

[23] Dodou E, Treisman R. 1997. The Saccharomyces cerevisiae MADS-box transcription factor Rlm1 is a target for the Mpk1 mitogen-activated protein kinase pathway. Mol Cell Biol 17:1848–1859. doi:10.1128/MCB.17.4.1848 9121433 PMC232032

[24] Jung US, Levin DE. 1999. Genome-wide analysis of gene expression regulated by the yeast cell wall integrity signalling pathway. Mol Microbiol 34:1049–1057. doi:10.1046/j.1365-2958.1999.01667.x 10594829

[25] Gilmore SA, Voorhies M, Gebhart D, Sil A. 2015. Genome-wide reprogramming of transcript architecture by temperature specifies the developmental states of the human pathogen Histoplasma. PLoS Genet 11:e1005395. doi:10.1371/journal.pgen.1005395 26177267 PMC4503680

[26] Vienken K, Fischer R. 2006. The Zn(II)2Cys6 putative transcription factor NosA controls fruiting body formation in Aspergillus nidulans. Mol Microbiol 61:544–554. doi:10.1111/j.1365-2958.2006.05257.x 16780567

[27] Schütte M, Thullier P, Pelat T, Wezler X, Rosenstock P, Hinz D, Kirsch MI, Hasenberg M, Frank R, Schirrmann T, Gunzer M, Hust M, Dübel S. 2009. Identification of a putative Crf splice variant and generation of recombinant antibodies for the specific detection of Aspergillus fumigatus. PLoS One 4:e6625. doi:10.1371/journal.pone.0006625 19675673 PMC2721682

[28] Assa D, Voorhies M, Sil A. 2025. Chemical stimuli override a temperature-dependent morphological program by reprogramming the transcriptome of a fungal pathogen. mBio 16:e02234-25. doi:10.1128/mbio.02234-25 40928299 PMC12505909

[29] Longo LVG, Ray SC, Puccia R, Rappleye CA. 2018. Characterization of the APSES-family transcriptional regulators of Histoplasma capsulatum. FEMS Yeast Res 18:foy087. doi:10.1093/femsyr/foy087 30101348 PMC6454542

[30] Rocafort M, Fudal I, Mesarich CH. 2020. Apoplastic effector proteins of plant-associated fungi and oomycetes. Curr Opin Plant Biol 56:9–19. doi:10.1016/j.pbi.2020.02.004 32247857

[31] Rodriguez RA, Azimova D, Voorhies M, English BC, Symington J, Sil A. 2025. Expansion of secreted cysteine knot proteins reveals virulence factors in the human fungal pathogen Histoplasma. Cell Rep 44:116465. doi:10.1016/j.celrep.2025.116465 41138184

[32] Youseff BH, Holbrook ED, Smolnycki KA, Rappleye CA. 2012. Extracellular superoxide dismutase protects Histoplasma yeast cells from host-derived oxidative stress. PLoS Pathog 8:e1002713. doi:10.1371/journal.ppat.1002713 22615571 PMC3355102

[33] Ivey FD, Magee DM, Woitaske MD, Johnston SA, Cox RA. 2003. Identification of a protective antigen of Coccidioides immitis by expression library immunization. Vaccine (Auckl) 21:4359–4367. doi:10.1016/s0264-410x(03)00485-7 14505918

[34] Belaish R, Sharon H, Levdansky E, Greenstein S, Shadkchan Y, Osherov N. 2008. The Aspergillus nidulans cetA and calA genes are involved in conidial germination and cell wall morphogenesis. Fungal Genet Biol 45:232–242. doi:10.1016/j.fgb.2007.07.005 17703972

[35] Isaac DT, Berkes CA, English BC, Murray DH, Lee YN, Coady A, Sil A. 2015. Macrophage cell death and transcriptional response are actively triggered by the fungal virulence factor Cbp1 during H. capsulatum infection. Mol Microbiol 98:910–929. doi:10.1111/mmi.13168 26288377 PMC5002445

[36] Camilli G, Blagojevic M, Naglik JR, Richardson JP. 2021. Programmed cell death: central player in fungal infections. Trends Cell Biol 31:179–196. doi:10.1016/j.tcb.2020.11.005 33293167 PMC7880884

[37] Lange T, Kasper L, Gresnigt MS, Brunke S, Hube B. 2023. “Under pressure” - how fungi evade, exploit, and modulate cells of the innate immune system. Semin Immunol 66:101738. doi:10.1016/j.smim.2023.101738 36878023 PMC10109127

[38] Benson S, Anderson CJ. 2026. Dead but not gone: the interplay between the programmed cell death process and surrounding bacteria. Infect Immun 94:e00509-24. doi:10.1128/iai.00509-24 41563099 PMC12890029

[39] Odio CD, Marciano BE, Galgiani JN, Holland SM. 2017. Risk factors for disseminated coccidioidomycosis, United States. Emerg Infect Dis 23:308–311. doi:10.3201/eid2302.160505 28098554 PMC5324825

[40] Cohen A, Jeng EE, Voorhies M, Symington J, Ali N, Rodriguez RA, Bassik MC, Sil A. 2022. Genome-scale CRISPR screening reveals that C3aR signaling is critical for rapid capture of fungi by macrophages. PLoS Pathog 18:e1010237. doi:10.1371/journal.ppat.1010237 36174103 PMC9578593

[41] Isaac DT, Coady A, Van Prooyen N, Sil A. 2013. The 3-hydroxy-methylglutaryl coenzyme A lyase HCL1 is required for macrophage colonization by human fungal pathogen Histoplasma capsulatum. Infect Immun 81:411–420. doi:10.1128/IAI.00833-12 23184522 PMC3553812

[42] de Melo Teixeira M, Stajich JE, Sahl JW, Thompson GR, Brem RB, Dubin CA, Blackmon AV, Mead HL, Keim P, Barker BM. 2022. A chromosomal-level reference genome of the widely utilized Coccidioides posadasii laboratory strain “Silveira”. G3 12:jkac031. doi:10.1093/g3journal/jkac031 35137016 PMC8982387

[43] Li B, Dewey CN. 2011. RSEM: accurate transcript quantification from RNA-Seq data with or without a reference genome. BMC Bioinformatics 12:323. doi:10.1186/1471-2105-12-323 21816040 PMC3163565

[44] Bray NL, Pimentel H, Melsted P, Pachter L. 2016. Near-optimal probabilistic RNA-seq quantification. Nat Biotechnol 34:525–527. doi:10.1038/nbt.3519 27043002

[45] Lun ATL, Chen Y, Smyth GK. 2016. It’s DE-licious: a recipe for differential expression analyses of RNA-seq experiments using quasi-likelihood methods in edgeR. Methods Mol Biol 1418:391–416. doi:10.1007/978-1-4939-3578-9_19 27008025

[46] de Hoon MJL, Imoto S, Nolan J, Miyano S. 2004. Open source clustering software. Bioinformatics 20:1453–1454. doi:10.1093/bioinformatics/bth078 14871861

