# Supplementary figures and images for: Macrophage phagocytosis of Coccidioides promotes its differentiation into the parasitic form
Source: mBio. 2026 May 18;17(6):e00492-26. doi: 10.1128/mbio.00492-26 (PMC13251436; doi:10.1128/mbio.00492-26)

**S1 Fig**

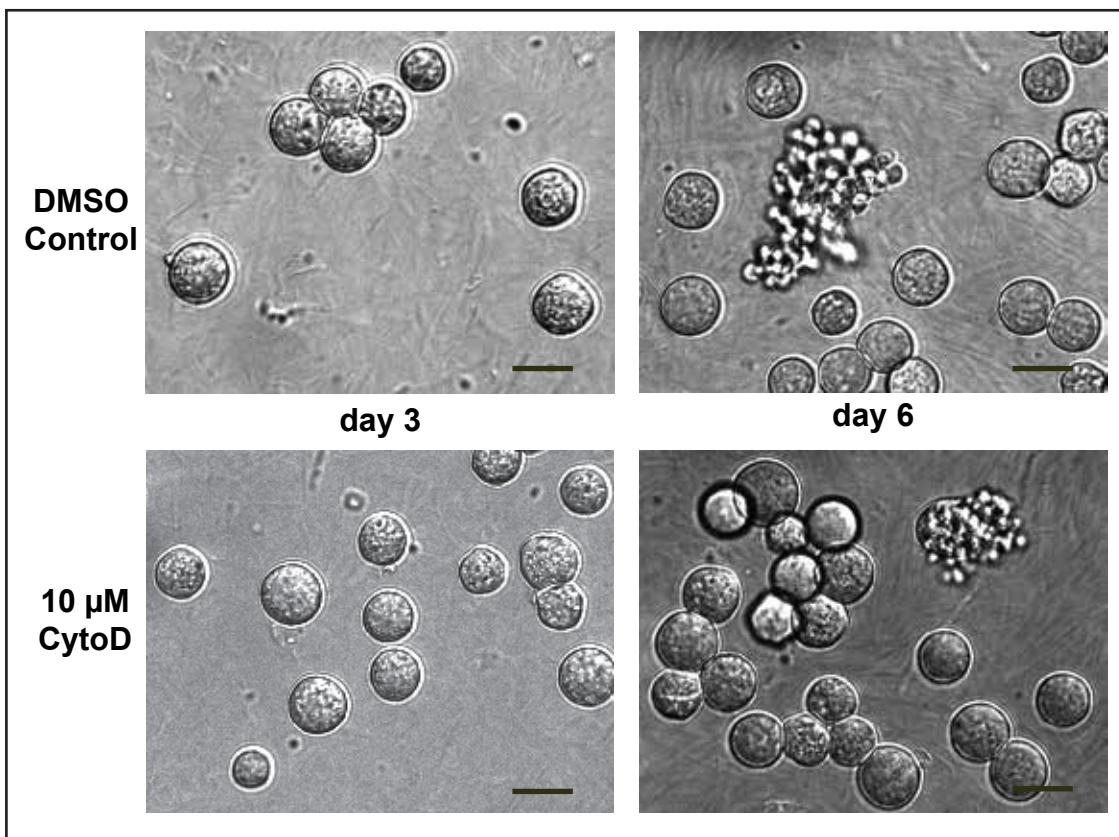

S2 Fig

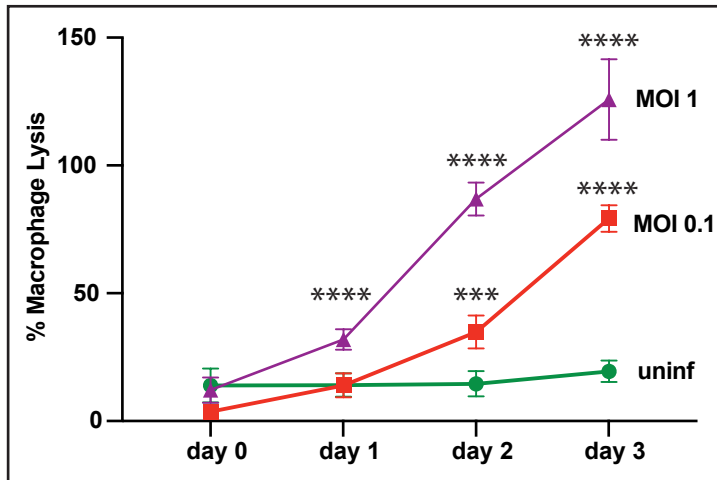

Supplement: Supplemental figures — Figures S1 and S2. [file mbio.00492-26-s0001.pdf]
