# Supplementary material for: Macrophage phagocytosis of Coccidioides promotes its differentiation into the parasitic form
Source: mBio. 2026 May 18;17(6):e00492-26. doi: 10.1128/mbio.00492-26 (PMC13251436; doi:10.1128/mbio.00492-26)
Supplement: Supplemental Legends — Legends for supplemental videos, figures, and tables. [file mbio.00492-26-s0002.docx]

**Supplemental Legends**

**Video S1. *Coccidioides* arthroconidia grow primarily as hyphae in tissue culture conditions.** Representative video of *Coccidioides* arthroconidia grown in BMM at 37 ˚ C with 5% CO_2_ with images taken every 1 hour over the course of 3 days. Arthroconidia germinate to mostly become hyphae with one spherule in the frame.

**Video S2. *Coccidioides* arthroconidia grown in the presence of macrophages develop into spherules.** Representative video of *Coccidioides* arthroconidia grown in the presence of BMDM in BMM at 37 ˚ C with 5% CO_2_ with images taken every 1 hour over the course of 3 days. Arthroconidia are associated with macrophages and become spherules up to 40 µm in diameter with hyphae entering the field at later timepoints.

**Fig S1. Cytochalasin D does not prevent spherule formation under in vitro spherulation conditions.** *Coccidioides* arthroconidia were grown in Converse media at 39 ˚ C with 10% CO_2_ in the presence of 10 µM cytochalasin D or DMSO for 6 days. Pictures were taken on days 3 and 6. Data presented was representative of 3 independent flasks for each condition in 2 independent experiments. Scale bar represents 10 µm.

**Fig S2. *Coccidioides* arthroconidia induce BMDM lysis.** BMDMs were infected with *Coccidioides* arthroconidia at an MOI of either 1 or 0.1. Lactate dehydrogenase (LDH) assay was used to measure % BMDM lysis over three days. All timepoints are relative to timepoint zero of uninfected cells lysed with 1% Triton-X (total LDH). The total LDH at later timepoints can be greater than the total LDH from the initial timepoint due to continued replication of BMDMs over the course of the experiment, resulting in an apparent lysis that is greater than 100%. Data presented are representative of 2 independent experiments. T-test comparing each point to uninfected at same time point, *** p ≤ 0.001, **** p ≤ 0.0001.

**Table S1.  Transcript abundances and differential expression of *Coccidioides* grown alone or in the presence of BMDMs.** Excel-compatible tab-delimited text conforming to Java TreeView extended CDT format.  Each row is a transcript, with the UNIQID column giving the *C. posadasii* Silveira systematic gene name from the PRJNA664774 reference genome.  The NAME column gives short names taken from Table S1 of Mandel et al (14). The next columns give edgeR BH-adjusted p-values for differential expression in each of the 7 contrasts.  The next 6 columns give systematic names and annotations for related genomes as mapped by INPARANOID in Mandel et al (14): previous *C. posadasii* Silveira annotation (CpV2 and Cp_anno), *C. immitis* RS (CiRS and CiRS_anno) and *H. ohiense* G217B (HcG217B and HcG217B_anno).  The next 24 columns give integer-rounded KALLISTO estimated counts for each transcript in each sample.  The counts are followed by a GWEIGHT place-holder column for Java TreeView compatibility.  The final 7 columns give the edgeR fit log2 ratios for each contrast.  The estimated counts in this file are sufficient to recapitulate the edgeR analysis.
**Table S2.  Transcripts differentially expressed in at least one condition.**  Excel-compatible tab-delimited text conforming to Java TreeView extended CDT format. Columns are as in Table S1, dropping the KALLISTO estimated count columns, with rows restricted to the 3,753 transcripts differentially expressed in at least one condition.
**Table S3.  Comparative profiles of *Coccidioides* germinating in the presence of macrophages or in vitro.**  Excel-compatible tab-delimited text conforming to Java TreeView extended CDT format.  Each row is a transcript, with the UNIQID column giving the *C. posadasii* Silveira systematic gene name from the PRJNA664774 reference genome.  Annotation columns are as in Table S1.  The first six contrasts are taken from the expression profiling of Table S1.  The final three columns are taken from the supplement of Homer et al., 2025 (6).
**Table S4.  Core spherule-enriched transcripts.**  Excel-compatible tab-delimited text conforming to Java TreeView extended CDT format. Columns are as in Table S3 with rows restricted to the 143 core spherule-enriched transcripts (green set in Fig. 5B and 5C).
**Table S5.  Macrophage-specific spherule-enriched transcripts.**  Excel-compatible tab-delimited text conforming to Java TreeView extended CDT format. Columns are as in Table S3 with rows restricted to the 229 macrophage-specific transcripts (orange set in Fig. 5B and 5C).

**Table S6.  Candidate secreted effectors.** Excel-compatible tab-delimited text.  Each row is a transcript.  First two columns give systematic ID and short name as in Table S1.  Next two columns give the number of cysteines and total number of amino acids in the predicted protein product.  Fifth column ("passes criteria") is True for < 250 amino acids and ≥ 4 cysteines and False otherwise.  Remaining annotation columns are taken from Table S1.
